# Transvaginal Repair of Supratrigonal, Juxtacervical, Vault, and Apical Vesicovaginal Fistulae: A Systematic Review and Meta-Analysis

**DOI:** 10.5152/tud.2025.24184

**Published:** 2025-06-24

**Authors:** Mugdha Srivastava, Ankur Mittal, Vikas Kumar Panwar, Yogesh Bahurupi, Arup Kumar Mandal

**Affiliations:** 1 Department of Urology, All India Institute of Medical Sciences, Rishikesh, India; 2 Department of Community and Family Medicine, All India Institute of Medical Sciences, Rishikesh, India

**Keywords:** Supratrigonal VVF, vaginal repair, vesicovaginal fistula

## Abstract

**Objective:**

This systematic review and meta-analysis assesses the success rate, surgical and quality of life outcomes, and complications of vaginal repair of supratrigonal, juxtacervical, vault, and apical vesicovaginal fistulae (VVF) as a group.

**Methods:**

PubMed, Embase, and Cochrane were searched for studies published from January 2003 to August 2023. Sixteen (n = 612) and 15 (n = 568) studies were included in the review and meta-analysis, respectively. Risk of bias assessment was done using the Methodological Index for Non-Randomized Studies (MINORS) criteria. Four studies (n = 196) reported sexual health outcomes. Comprehensive meta-analysis software (trial version 3) was used for quantitative synthesis.

**Results:**

The success rate (95% CI) of vaginal repair of this specific group of VVF using a random effects model was 86.3% (76.5%-92.4%). *I*^2^ was 73.72% with a Q-value of 53.27. The mean age of patients was 43.7 years. Follow-up duration ranged from 1 to 84 months. There were no major intraoperative complications except for 1 inadvertent bowel injury. Postoperative complications included Urinary tract infections (n = 5), stress incontinence (n = 2), urge incontinence (n = 2), hematuria (n = 3), and vaginal bleeding (n = 3). One hundred eighty-four patients reported no sexual dysfunction, while 6 patients had a Female Sexual Function Index score ≤ 26.5.

**Conclusion:**

The studies included in this meta-analysis are largely heterogeneous and retrospective, which is a limitation of this meta-analysis. Despite this, the results of this meta-analysis confirm successful correction of most of the VVF included in this review by the transvaginal route. While preferences for a given surgical approach may vary based on the number and size of the VVF or vaginal capacity, these factors need to be studied prospectively to understand their role in deciding the route of repair.

**Main Points**

- Most supratrigonal, juxtacervical, vault, and apical vesicovaginal fistulae (VVF) can be successfully repaired vaginally.
- Random effect analysis revealed the success rate of vaginal repair in these VVF to be 86.3% (76.5%-92.4%).
- The studies included in this review reported almost no intraoperative complications.
- The choice of route of repair of these VVF is currently mostly a matter of personal preference for the surgeons.
- Prospective studies may devise an assessment for choosing the route of repair for such VVF and the factors that facilitate vaginal repair of these VVF.

## Introduction

Vesicovaginal fistula (VVF) is defined as “an abnormal communication between the bladder and the vagina, resulting in continuous involuntary discharge of urine through the vagina.” It is a distressing condition for patients and profoundly impacts the quality of life. Even though rare in the developed world, the most common cause in developed countries is gynecological surgeries, e.g., hysterectomies, which account for 80% of the new cases in these countries. However, in developing countries, the condition contributes greatly to morbidity, with obstructed labor being the most common cause.^1^ It is estimated that around 30 000-130 000 new cases of VVF occur each year in the African continent alone, and that at least 3 million women have unrepaired VVF in underdeveloped countries.^2^ Despite the huge burden of the disease, the true global incidence remains hidden due to the stigma surrounding the condition.^1^ Finding the incidence and prevalence of supratrigonal, vault, apical, and juxtacervical VVF poses a bigger challenge still. There is a dearth of evidence on the epidemiology of these VVFs.

The management options for VVF vary from conservative to surgical. Unfortunately, conservative methods fail often leaving surgical repair as the only option.^2^^,^^3^ A myriad of factors affect the choice of surgical route of repair; surgeon’s preference or familiarity, space in the vaginal cavity, fistula location, need for concurrent procedures, and the accessibility of interposition grafts, etc.^1^^,^^4^ For example, most gynecologists prefer the vaginal route of repair which might offer some advantages over the abdominal approach.^2^^-^^5^ Vaginal route might minimize the operative complications such as hospital stay and blood loss, but, it is also believed to be associated with vaginal shortening, which in turn is believed to cause postoperative morbidity.^2^ The factors that deter surgeons from choosing this route may include a poorly compliant or a small bladder, the need for a simultaneous ureteral reimplantation, or vaginal stenosis.^2^ However, there have been no randomized trials comparing the abdominal route of repair with the vaginal route of repair to date.

This systematic review and meta-analysis aims to study the current literature on vaginal surgical repair of supratrigonal, juxtacervical, vault, and apical VVF as a group with emphasis on its success rate and the surgical and quality of life outcomes to synthesize consolidated evidence on the vaginal repair of these VVF. By doing so, this review and meta-analysis aims to not only identify the existing gaps in knowledge but also to provide insights that can guide future research endeavours and inform evidence-based practices.

## Materials and Methods

### Outcome Measures

Primary outcome: To assess the success rate of the vaginal route of repair of supratrigonal, juxtacervical, vault, and apical VVF as a group.

Secondary outcomes: (i) To assess the surgical and quality of life outcomes of the vaginal route of repair in this specific group of VVF, (ii) to assess the factors that promote the vaginal route of repair of these VVF, and (iii) to assess the complications of the vaginal route of repair of these VVF.

### Search Strategy

This review and meta-analysis is reported in accordance with the Preferred Reporting Items for Systematic Review and Meta-analysis (PRISMA) guidelines and was approved by the Institutional Ethics Committee. The protocol was registered in the International Prospective Register of Systematic Reviews (PROSPERO) (ID: CRD42023471585).

For this review and meta-analysis, a group of VVF which includes supratrigonal VVF, juxtacervical VVF, vault VVF, and apical VVF have been studied. From here onwards, “VVF” will refer to one of these kinds of VVF (supratrigonal VVF/juxtacervical VVF/vault VVF/apical VVF) unless specified. A comprehensive literature search was performed in PubMed, Embase, and Cochrane databases using a combination of the following search terms: “vesicovaginal fistula,” “supratrigonal vesicovaginal fistula,” “high vesicovaginal fistula,” “juxtacervical vesicovaginal fistula,” “vault vesicovaginal fistula,” “apical vesicovaginal fistula,” “urogenital fistula,” “gynecologic surgical procedures,” “urologic surgical procedures,” “latzko repair,” “modified latzko repair,” “transvaginal repair,” and “latzko technique.” The search strategy for each database is given in detail in Table 1. Abstract screening was followed by full-text screening. The search was limited to the period between January 2003 and August 2023. Searches for all 3 databases were performed between September 2023 and October 2023. Conference abstracts and abstracts for which full-text articles were unavailable were excluded. Studies not in the English language, case reports, and case series having fewer than 3 cases were also not included in the review and meta-analysis. Additional hand searches were performed in the relevant articles.

### Evidence Synthesis

A systematic review was conducted, and the studies were included or excluded based on a set of predefined criteria. Full-text articles reporting the vaginal route of repair in supratrigonal, juxtacervical, vault, and apical VVF were included. Studies reporting genitourinary fistulae other than these VVF or reporting management other than vaginal route of repair were excluded. Four hundred and seven studies were screened after removing duplicates. Of those, 95 were evaluated for full-text eligibility. Twenty-three reports needed further elucidation of their data for the purposes of this review, and the authors of all 23 articles were sent an email requesting additional data. Panaiyadiyan et al^6^ and Lee et al^7^ sent the data and these were included in the review. Sixteen studies measured and reported the outcomes of interest and were finally included in the review, and out of these 16, 15 studies were included in the meta-analysis.

**Figure 1.**
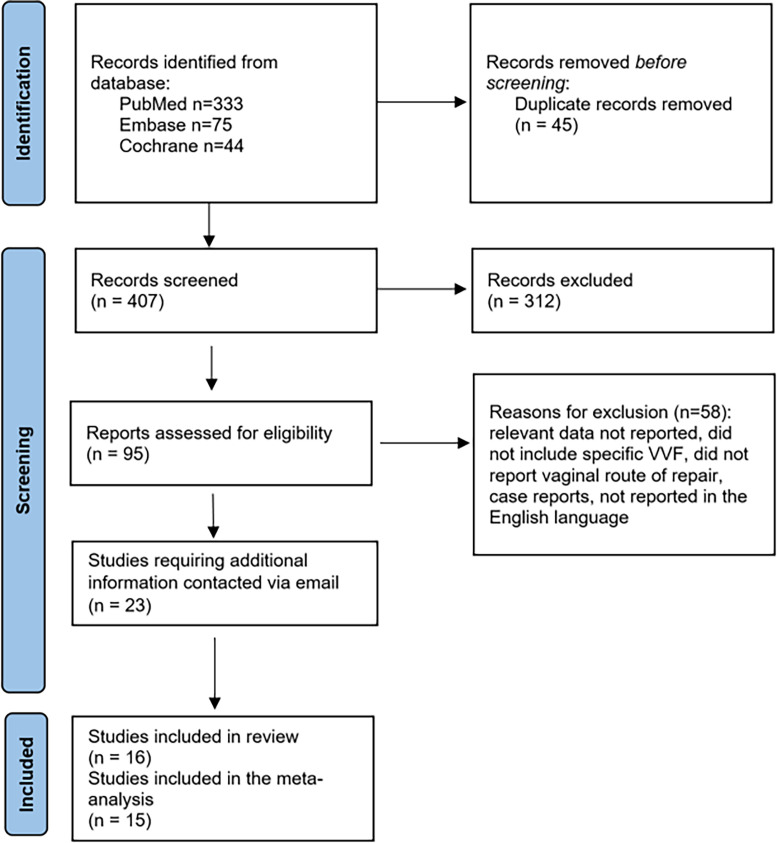
Preferred Reporting Items for Systematic Review and Meta-analysis flow diagram for study selection.

The PRISMA flowchart illustrating the literature search and selection of eligible studies is given in Figure 1. The titles and abstracts were screened independently by 2 reviewers (M.S. and A.M.). Any disagreements were resolved by a third reviewer (V.K.P.). Review of full text as well as data extraction followed the same pattern. The extracted data included, among other details, the following: year of publication, study design, demography, sample size, success rate of the procedure, details of flaps used, operative time, blood loss, duration of hospital stay, and follow-up, etc.

### Quality Assessment

Two independent reviewers (M.S. and A.M.) used the Methodological Index for Non-Randomized Studies (MINORS) to assess the quality of each study included in the review.^8^ Any disagreements were resolved by a third reviewer (V.K.P.). The original tool was modified based on the studies included in this review and included 5 items instead of the original 8 for non-comparative studies, except for 2 studies where the data were prospectively collected and thus included all 8 items.^9^ Each item was assigned a score as follows: 0 (for not reported), 1 (if reported but inadequate), or 2 (reported and adequate). The total score for a study was the sum of all the scores for each item. The risk of bias was then graded as follows: (i) for the retrospective and cross-sectional studies: 7-10 (low risk of bias), 4-6 (intermediate risk of bias), and 1-3 (high risk of bias) and (ii) for the 2 studies with prospective data collection: 11-16 (low risk of bias), 6-10 (intermediate risk of bias), and 1-5 (high risk of bias).

### Statistical Analysis

The analysis is based on 15 studies. The outcome was the success rate of vaginal repair. The meta-analysis was performed using both the fixed effects and random effects models. The Q statistic test and *I*^2^ statistic were performed to evaluate heterogeneity. All analyses were done using Comprehensive Meta-Analysis software (version 3.0, trial version).^10^ Forest plots were created and the results reported along with the Q-test, *I*^2^, and 95% confidence intervals of the outcome in the pooled analysis. Mean (SD) was reported whenever available. If studies reported means without SDs, they were presented as reported.

## Results

### Overall Study Characteristics

We included 16 studies with 612 cases in the systematic review and 15 studies with 568 cases in the meta-analysis. The characteristics of individual studies are given in Table 2.

### Quality Assessment

Nine studies were found to have a low risk of bias, while 5 had an intermediate risk of bias, as shown in Table 3a. The risk of bias in both the studies reported by Umoiyoho et al^20^ and Panaiyadiyan et al^6^ was low, as given in Table 3b.

### Success Rate of Vaginal Repair

Fifteen studies were included in the meta-analysis (Group 1). *I*^2^ for these 15 studies was 73.72% with a Q-value of 53.27. One study by Kizilay et al^11^ had a success rate that was vastly different from the rest of the studies in the analysis.^11^ On excluding this study, the *I*^2^ for the group (Group 2) came down to 66.61% with a Q-value of 38.94. Both the fixed effects and random effects analyses for both these groups are given here.

- Random effects model

**Figure 2.**
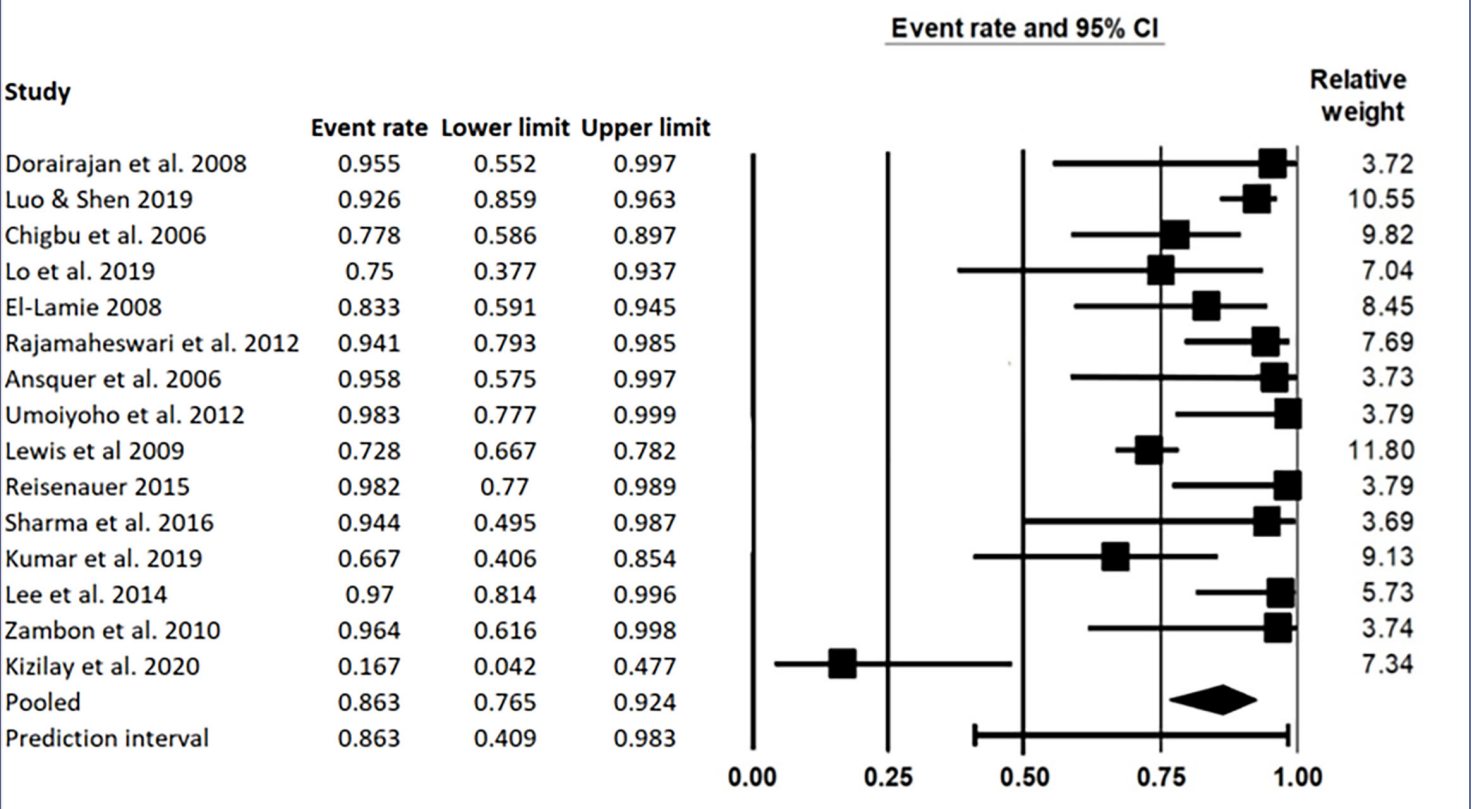
Success rate Group 1 - Random effects model, *I*^2^ = 73.72%, Q-value = 53.27.

**Figure 3.**
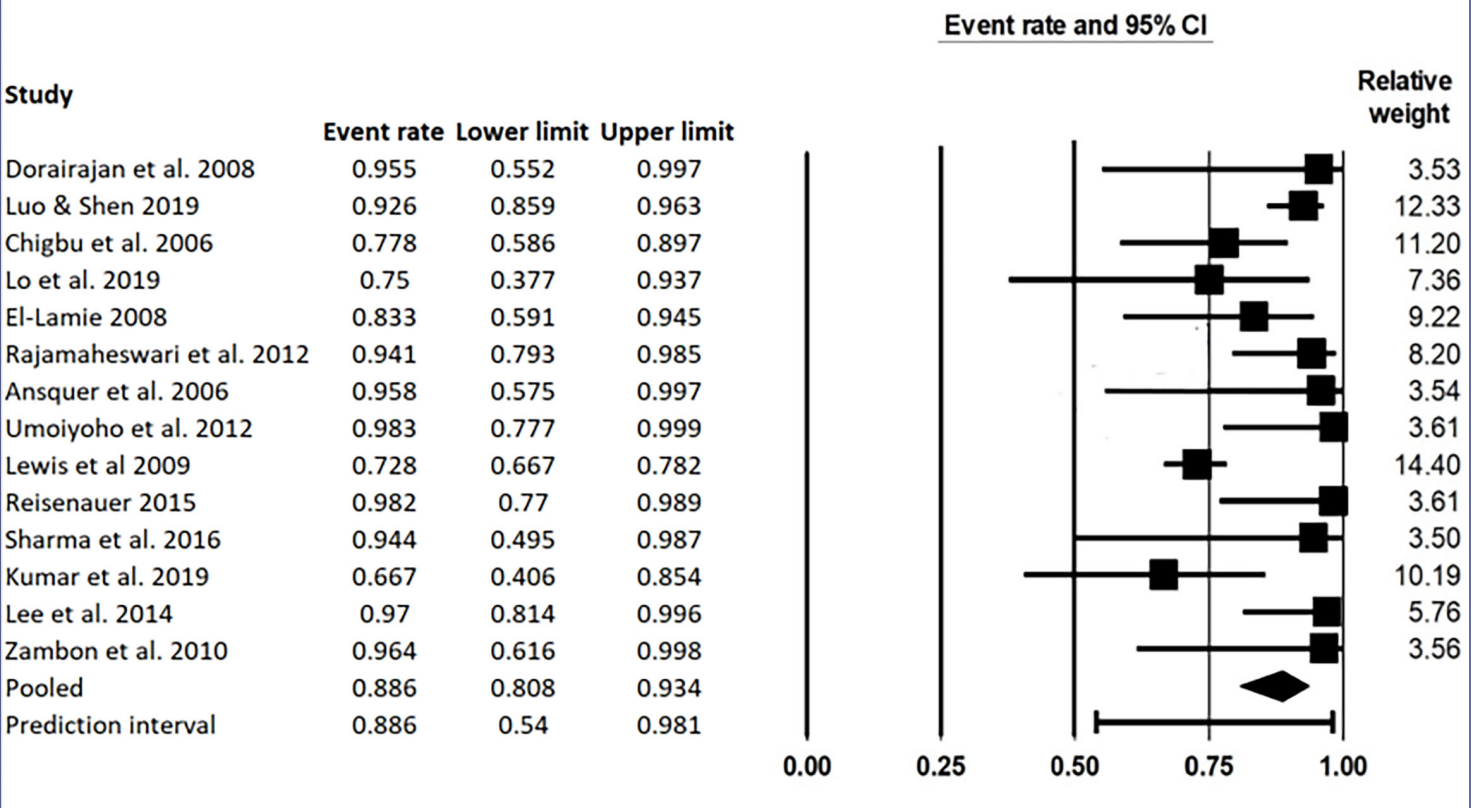
Success rate Group 2 - Random effects model, *I*^2^ = 66.61%, Q-value = 38.94.

The success rate (95% CI) of vaginal repair in Group 1 is 86.3% (76.5%-92.4%), Figure 2while the success rate in Group 2 was found to be 88.6% (80.8%-93.4%), Figure 3.

- Fixed effects model

Even though the studies in the analysis had high heterogeneity, the fixed effects model analysis has been reported here for the sake of completeness.

**Figure 4.**
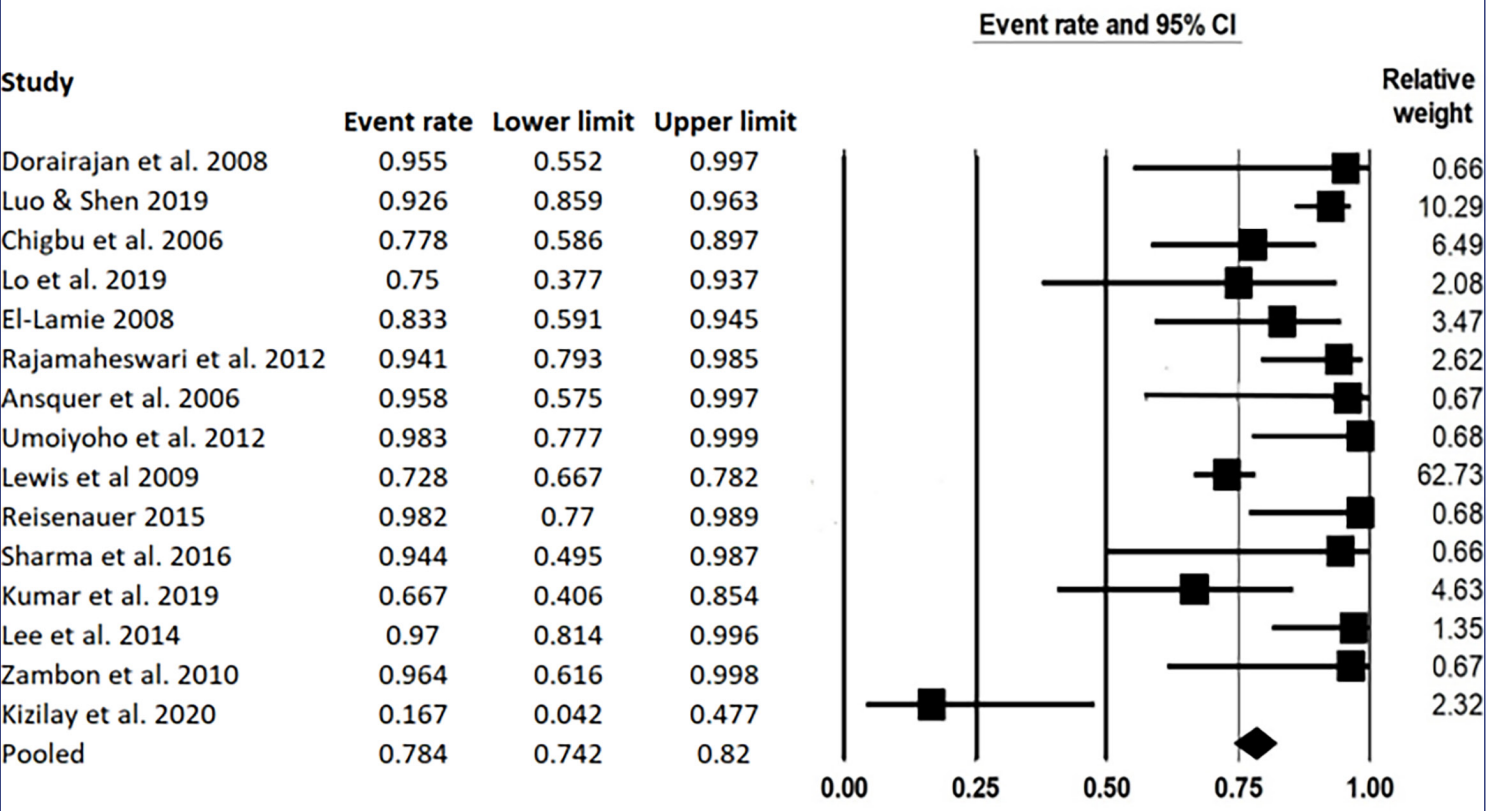
Success rate Group 1 - Fixed effects model, *I*^2^ = 73.72%, Q-value = 53.27.

**Figure 5.**
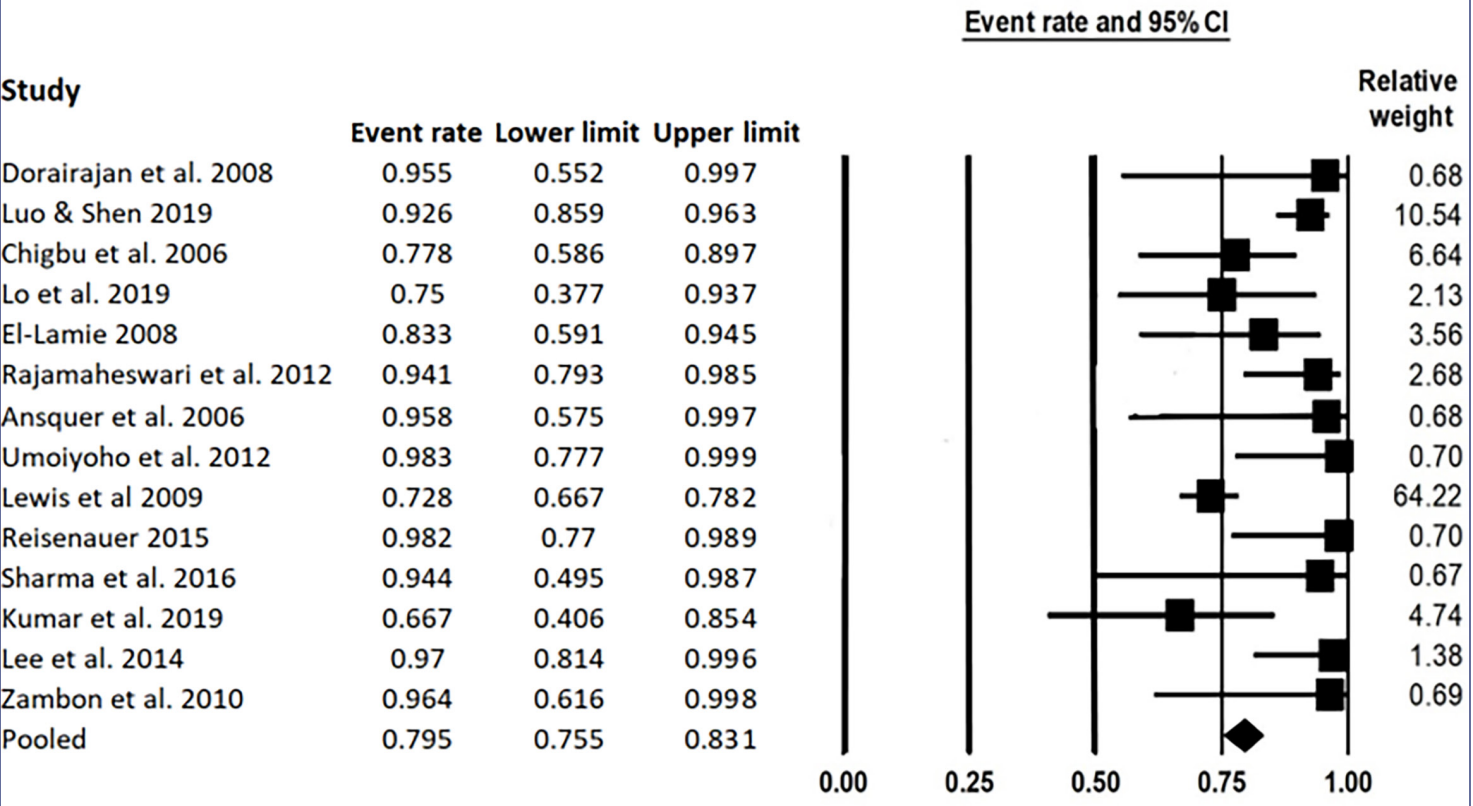
Success rate Group 2 - Fixed effects model, *I*^2^ = 66.61%, Q-value = 38.94.

The success rate (95% CI) of vaginal repair in Group 1 is 78.4% (74.2%-82%), Figure 4. While the success rate in Group 2 was found to be 79.5% (80.8%-93.4%), Figure 5.

### Secondary Outcomes

Surgical outcomes such as operative time, blood loss, need for blood transfusion, duration of hospital stay, and duration of insertion of postoperative catheter, along with quality of life outcomes and complications of vaginal route of repair, are given in detail in Table 4. Out of the reported quality of life outcomes by Dorairajan et al,^5^ 8 patients had resumed sexual activity without discomfort, as observed with a minimum follow-up of 2 years. Rajamaheswari et al^12^ reported no cases of dyspareunia, increased urinary frequency, or urgency. Lo et al^13^ reported a mean score of 9 on the Urogenital Distress Inventory (UDI-6) and a mean score of 8.75 on the Incontinence Impact Questionnaire (IIQ-7). Luo and Shen reported no sexual discomfort in any of the patients post-repair while reporting “very much better” in 53.7% of their cohort on the Perception Global Impression of Improvement questionnaire (PGI-I), while the rest of the cohort reported “better.”^14^ Panaiyadiyan et al^6^ reported a Female Sexual Function Index (FSFI) score of ≤ 26.5 in 6 patients. Four patients had an International Consultation of Incontinence Questionnaire - Short Form (ICIQ-SF) score of 4, while for the rest, it was 0. Urge incontinence developed in 2 patients in their cohort.^6^ Among all the studies included in this review, UTI was reported in n = 5, stress urinary incontinence in n = 2, while Rajamaheswari et al^12^ reported 1 inadvertent bowel injury.^12^ Chigbu et al^15^, Zambon et al,^16^ and Kizilay et al^11^ reported no complications.

The choice of route of repair was based mostly on the surgeon’s discretion in the studies that were reviewed. However, the following factors were found to impede vaginal repair and prompted abdominal repair when present: inadequate exposure because of anatomy, close proximity to the ureteral orifices, and requirement of ureteral reimplantation. Table 5 gives an extensive list.

## Discussion

This systematic review and meta-analysis aimed to assess the success rate of vaginal repair of supratrigonal, juxtacervical, vault, and apical VVF, as well as its surgical and quality of life outcomes, complications, and the factors that promote vaginal repair of these VVF.

To the best of the authors’ knowledge, this is the first such systematic review and meta-analysis. There is a dearth of literature on vaginal repair of these VVFs. It was found that most such VVFs are amenable to successful vaginal repair with minimal perioperative and postoperative complications. It was also found that, contrary to popular opinion, most vaginal repairs did not lead to sexual dysfunction.

Chigbu et al^15^ reported a multicentre study. The reason for the failure of the vaginal repair in their cohort was found to be difficult access in all 6 patients. All 6 patients had a second successful repair, but it was done abdominally this time.^15^ Ansquer et al^17^ studied the Latzko procedure in vault VVF performed by 3 different surgeons. The mean body mass index (BMI) of their population was 24 kg/m^2^. Two of their patients had previously undergone fistula repair via other techniques, yet the Latzko technique proved to be successful when the authors operated. Thus, they concluded that a previous repair via any technique is not a contraindication for the Latzko procedure.^17^ Chigbu et al^15^ decided on the route of repair after examination under anesthesia but did not mention the exact factors that prompted vaginal repair over abdominal repair. However, the authors concluded that the choice of route of repair should be individualized based on the accessibility of the fistula as examined under anesthesia. Interestingly, they did not find the size of the fistulae to be significantly different between the patients operated on via the abdominal and the vaginal routes in their population.^15^ Rajamaheswari et al,^12^ like Chigbu et al,^15^ examined all patients under anesthesia for site, size, number of fistulae, vaginal mobility, and fibrosis surrounding the fistula. They combined it with cystoscopy to confirm their findings. They chose the vaginal route if the vaginal wall was mobile and the pelvic floor relaxed. However, 14 patients were excluded from undergoing vaginal repair and underwent abdominal repair for the following reasons: fistula too high to reach vaginally, restricted vagina, ureteral reimplantation required, or co-morbidities requiring open surgery.^12^ Two cases reported by Rajamaheswari et al^12^ failed the initial vaginal repair and presented with urinary leakage on the 16th and 30th postoperative days. One of them underwent a successful second vaginal repair, while the other had to undergo abdominal repair since it required ureteral reimplantation. The one patient who had a bowel injury had previously undergone 3 abdominal surgeries, and postoperative bowel adhesions probably contributed to the event. The authors concluded that all supratrigonal VVFs do not necessarily require an abdominal approach just because they are located high up and that nearly 75% of gynecological supratrigonal VVF can show a success rate comparable to abdominal repair when repaired vaginally.^12^ Similarly, Dorairajan et al^5^ concluded that post-hysterectomy vault fistulae are esp. amenable to vaginal repair since their usual characteristics such as, a supratrigonal, single fistula, situated away from the ureteric orifices and on the vault with the posterior edge of the fistula corresponding to the vault scar, aid in preventing the inclusion of ureters while suturing even without opening the bladder. It even maintains vaginal depth despite the partial colpocleisis, contrary to popular belief.^5^

El-Lamie preferred to repair the fistula early in the course, ie, within 6 weeks of diagnosis, except in 3 cases in which the patients had received adjuvant radiotherapy, which were operated on nearly 6 months later. The repair in these 3 cases was delayed to give time to identify the full extent of the fistula after devascularisation. They had 3 cases of recurrence which were later managed successfully by the O’Connor technique with omental interposition.^18^

Zambon et al^16^ studied the vaginal and abdominal repair of complex supratrigonal VVF, the inclusion criteria being of note; size of fistula more than 2 cm, history of radiotherapy, concomitant ureteral fistulae and infection/laceration at the fistula site. Despite this, their success rate with vaginal repair was 100%. They set out to demonstrate that the vaginal approach was as effective as robotic or laparoscopic approaches, as minimally invasive, yet has a better learning curve, is more cost effective, and offers other advantages such as shorter hospital stays and faster recovery. In accordance with Sharma et al,^19^ they, thus, advocated for the need to keep this practice alive.^16^ Umoiyoho et al^20^ did a prospective study wherein they assessed the efficacy of a scoring system devised to sort patients of VVF repair based on the requirement of expertise in repair. All the repairs were done by a single surgeon using the same technique in hospital-based outreach programs. Patients without any urinary incontinence at the 6-week follow-up were considered to have been successfully repaired. Based on the scoring system, only relatively simple obstetric fistulae were included in the study, while complex fistulae were excluded and referred elsewhere since they required advanced care. The authors concluded that the scoring system effectively assesses simple VVFs. These fistulae, in turn, can be effectively repaired vaginally with a whopping success rate. Since this study included VVFs other than supratrigonal, juxtacervical, vault, and apical fistulae as well, however, the other findings in this population, such as age, parity, and level of education were unable to be reported.^20^

Reisenauer reported transvaginal multilayer closure in 27 supratrigonal VVF, all done by the same surgeon, with a 100% success rate. They primarily chose vaginal repair because of its minimally invasive nature and better morbidity profile.^21^ Kumar et al^22^ studied transvaginal repair of VVF, including supratrigonal, subtrigonal, and urethrovaginal VVF. On multivariate regression analysis, they found fistula location to be one of the factors affecting outcome (OR 2.5), other factors being the underlying etiology and history of previous repair. The authors concluded as well that all vaginally accessible fistulae should be repaired via the vaginal route regardless of etiology.^22^

The majority of patients with a supratrigonal VVF in a retrospective case series by Lo et al^13^ were repaired via the vaginal route. The mean BMI of their cohort was 23.51 kg/m^2^. The mean distance of the fistulae from a ureteric orifice in their cohort was 1.69 cm. Along with blood loss, they also noted the postop drop in Hb, which was found to be 0.84 g/dL (mean). There were 2 cases of recurrence, which were successfully closed by a urologist via the abdominal route.^13^

Interestingly, Luo and Shen surmised that Latzko repair might be difficult for apical fistulae because of firm vaginal transverse scarring so they demonstrated a modification of the Latzko technique esp. for apical VVFs and VVFs with limited access. In this modification, they did not catheterise the fistula, contrary to popular practice, and the incision to reconstruct a new vaginal apex was made in the normal anterior and posterior vaginal epithelium, containing the entire transverse vaginal scar. Three to 4 layers of closure were made in the perivesical tissue. Like Rajamaheswari et al,^12^ they did not excise the fistulous tract.^14^ Patients can be discharged within 24 hours of this procedure with a Foley catheter left inserted for 4 weeks. Recurrence occurred in 8 patients of their cohort after the initial repair with the modified Latzko technique. However, all 8 of them had a successful closure with the same technique as well. Moreover, the authors reported the second repair was easier than the first, since the failed cases had a smaller fistula after the initial repair.^14^

Kizilay et al^11^ compared the Latzko repair with the abdominal bivalve technique in patients with supratrigonal, subtrigonal, and trigonal VVF. Even though the supratrigonal VVF in their population were more frequently repaired via the abdominal route, 12 supratrigonal VVF were still operated via the vaginal route with a recurrence rate of 83.3%, the highest recurrence rate for any study in this review. One reason given by the authors for this fact was that the majority of patients in their population had a previous history of radiotherapy.^11^ This is still in contrast to Zambon et al,^16^ who had a much higher success rate of 100% in their population with complex supratrigonal VVF.^11^

### Surgical and Quality of Life Outcomes

Among the population treated by Chigbu et al,^15^ Rajamaheswari et al,^12^ and Dorairajan et al,^5^ none of the patients who underwent vaginal repair required blood transfusion. All of the patients in the study done by Dorairajan et al^5^ were ambulatory on the first postoperative day. Contrary to popular belief, they did not find the shortening of the vagina to cause any functional disability. All 8 women living with their partners were able to resume sexual activity without any discomfort. Even though Dorairajan et al^5^ reported a prolonged hospital stay (more than 2 weeks) postoperatively, the authors suggested the patients could have been discharged earlier had it not been for their hospital policy, which did not allow patients to be discharged before catheter removal.^5^ Similarly, Sharma et al^19^ reported the outcomes of the Latzko procedure in 8 cases of supratrigonal VVF, all performed by the same surgeon with a 100% success rate. Even though their mean hospital stay was 2 weeks, the authors reported that the patients could have been discharged on postop day 5. The reason these patients were not discharged earlier was that they had all travelled to the hospital from far and wide and did not want to go back home before the removal of the catheter.^19^ Panaiyadiyan et al^6^ compared the quality of life outcomes between the transabdominal and the transvaginal repair of trigonal as well as supratrigonal VVF. However, unpublished data has been included in this review, since this review focuses on the vaginal repair of supratrigonal, juxtacervical, vault, and apical VVF. Seven out of the 44 women in their cohort were not sexually active before the development of VVF; 5 of those continued to be inactive even after the repair, and 1 had an FSFI score of 32.9 despite an ICIQ-SF score of 4 after the repair. Ten women reported sexual dysfunction, while 2 reported urinary dysfunction. Three of those patients avoided intercourse despite being completely cured for fear of urine leakage. This demonstrates the devastating impact of VVF on the psychology of patients. The cohort did not develop any major postoperative complications. The advantage this study provides over most others is its very long follow-up period, the mean follow-up period being 27.3 months. Thus, this study provides a much better perspective on how the vaginal route of repair affects the overall quality of life in the long run.^6^ In accordance with Dorairajan et al^5^ and Luo and Shen, they also did not find vaginal repair negatively affecting patients’ sexual health.^6^^,^^14^ They studied husband satisfaction scores in the evaluation of sexual function and found most partners to be either ‘moderately’ or ‘very’ satisfied. This shows that VVF repair has a very limited impact, if any, on the partner’s sexual satisfaction. The study has the following limitation: a few patients could not fill out the questionnaires on their own, and so a blinded co-investigator helped them. This does affect the quality of data.^6^

The clinical implications of the results of this meta-analysis are promising; for example, it was seen that the size of the fistulae were not a huge deterrence to vaginal repair, fistulae greater in size than even 2 cm can be successfully repaired vaginally, and supratrigonal fistulae located even high up can be successfully repaired vaginally.^12^^,^^15^^,^^16^ The study conducted by Zambon et al^16^ had inclusion criteria that are conventionally considered difficult for vaginal repair, yet, they reported a 100% success rate with vaginal repair. Moreover, the postoperative morbidity with vaginal repair was found to be low and recovery quicker. There were minimal postoperative complications. The patients in this review, by and large, did not seem to struggle with their sexual health in terms of functional ability postoperatively either.^5^^,^^6^^,^^14^

### Strengths and Limitations

We set out with clear and precise aims for the review and meta-analysis at the outset. Care was taken not to miss a single study of relevance. The evidence for this review and meta-analysis was gathered with a robust and exhaustive search strategy curated specifically for each database (Table 1). No stones were left unturned to gather more data, wherever applicable, from authors of each study that came up and was relevant to the review. Each study underwent a thorough and rigorous review and data collection process. However, this review and meta-analysis is limited by its exclusion of studies that were not published in the English language. The studies that did meet the inclusion criteria were vastly heterogeneous and retrospective. The potential reasons for this heterogeneity among the studies included in the review could be: the differences in the fistulae and patient characteristics (e.g., cultural and environmental), study design, outcome measures employed, postoperative care, follow-up duration, and selection bias while deciding the route of repair. As with all reviews and meta-analyses, this one is also limited by the inherent limitations of each study included in the review and has a potential for publication bias.

This review demonstrates that most supratrigonal, juxtacervical, vault, and apical VVF can be successfully repaired vaginally. A key point to note is the complete absence of any intraoperative complications, apart from the 1 inadvertent bowel injury reported by Rajamaheswari et al,^12^ in all the studies included in this review. The learning curve for Latzko repair is minimal.^5^ The historic lack of major complications such as bowel injury during the procedure and the absence of additional abdominal wounds postoperatively are an added advantage.^5^^,^^23^ The route of repair for most surgeons in the review was a matter of personal preference based on VVF location, complexity of the fistula, or involvement of other genitourinary structures.^6^^,^^7^ However, most surgeons preferred not to perform vaginal repair whenever ureteral reimplantation was required or if the fistula was in close proximity to the ureter. All studies except for the one reported by Umoiyoho et al^20^ were retrospective with a small sample size. Moreover, the studies were largely heterogeneous. Further research is needed, preferably with prospective design, to devise an objective assessment system for sorting supratrigonal, juxtacervical, vault, and apical VVF into 2 categories; those that can be repaired vaginally and those that cannot, and to study factors that facilitate vaginal repair of these VVF. Moreover, specific characteristics such as fistula location, patient’s age, surgical technique, etc., and their effects on the outcome of repair need to be studied. This was found to be a gap in existing knowledge. This review did not include a comparison between the vaginal repair with other modes that are currently employed by surgeons and further research can be done to compare the vaginal repair with other modes of repair in this group of VVF as well as comparing long-term outcomes of these different techniques.

## Figures and Tables

**Table 1.** Search Strategy

Database	Search Strategy	Filters Used	Results
PubMed	#1 “Vesicovaginal Fistula”[Mesh] OR “Vesicovaginal Fistula/prevention and control”[Mesh] OR “Vesicovaginal Fistula/surgery”[Mesh] OR “Vesicovaginal Fistula/therapy”[Mesh] OR “supratrigonal vesicovaginal fistula*”[tw] OR “high vesicovaginal fistula*”[tw] OR “juxtacervical vesicovaginal fistula*”[tw] OR “vault vesicovaginal fistula*”[tw] OR “apical vesicovaginal fistula*”[tw] OR “urogenital fistula*”[tw] OR “supra trigonal vesicovaginal fistula*”[tw] OR “supratrigonal vesicovaginal fistula*”[tw] OR “supra-trigonal vesicovaginal fistula*”[tw] #2 “Gynecologic Surgical Procedures”[Mesh] OR “Urologic Surgical Procedures”[Mesh] OR “latzko repair”[tw] OR “modified latzko repair”[tw] OR “transvaginal repair”[tw] OR “trans-vaginal repair”[tw] OR “latzko technique”[tw] OR “latzko operation”[tw] #1 AND #2	Text availability: Full text Publication date: January 1, 2003-August 18, 2023	N = 333
Embase	#1 “Vesicovaginal Fistula” OR “supratrigonal vesicovaginal fistula” OR “high vesicovaginal fistula” OR “juxtacervical vesicovaginal fistula” OR “vault vesicovaginal fistula” OR “apical vesicovaginal fistula” OR “urogenital fistula” OR “supra trigonal vesicovaginal fistula” OR “supratrigonal vesicovaginal fistula” OR “supra-trigonal vesicovaginal fistula” #2 “Gynecologic Surgical Procedures” OR “Urologic Surgical Procedures” OR “latzko repair” OR “modified latzko repair” OR “transvaginal repair” OR “trans-vaginal repair” OR “latzko technique” OR “latzko operation” #1 AND #2		N = 75
Cochrane	“Vesicovaginal Fistula” OR “supratrigonal vesicovaginal fistula” OR “high vesicovaginal fistula” OR “juxtacervical vesicovaginal fistula” OR “vault vesicovaginal fistula” OR “apical vesicovaginal fistula” OR “urogenital fistula” OR “supra trigonal vesicovaginal fistula” OR “supratrigonal vesicovaginal fistula” OR “supra-trigonal vesicovaginal fistula”	None	N = 44

**Table 2.** Characteristics of Studies

Study	Study Design	Country	Type of VVF	Surgical Procedure	Sample Size	Age Mean (range)	Etiology
Ansquer et al^17^ 2006	Retrospective	France	Vault	Latzko repair	11	50 years (37-68)	Hysterectomy n = 10
							Colpectomy and partial cystectomy due to malignancy n=1
Chigbu et al^15^ 2006	Retrospective review	Nigeria	Juxtacervical	Vaginal repair	27	_	Obstetric etiology
Dorairajan et al^5^ 2008	Retrospective review	India	Supratrigonal	Latzko repair	10	39 years (33-55)	Hysterectomy (due to uterine fibroids and dysfunctional uterine bleeding)
El-Lamie^18^ 2008	Retrospective	Egypt	Supratrigonal	Vaginal flap-splitting technique	18	_	Vaginal cerclage n = 2
							Hysterectomy (n = 12, with adjuvant radiotherapy n = 3)
							McIndoe vaginoplasty (n = 1)
Lewis et al 2009^24^	Retrospective analysis	Sierra Leone	Juxtacervical	Latzko repair	228	_	Obstetric fistulae
Zambon et al^16^ 2010	Retrospective	Brazil	Supratrigonal	Vaginal repair	13	_	_
Rajamaheswari et al^12^ 2012	Retrospective	India	Supratrigonal	Vaginal repair	34	38.12 years	Gynecological etiology
Umoiyoho et al^20^ 2012	Prospective	Nigeria	Juxtacervical	Vaginal repair under saddle block	28	_	Obstetric fistulae
Lee et al^7^ 2014	Retrospective	USA	Supratrigonal	Vaginal repair	33	_	Prior hysterectomy most common
Reisenauer^21^ 2015	Retrospective	Germany	Supratrigonal	Latzko repair and a multilayered closure	27	45.67 years (12-65)	Hysterectomy (Lap assisted vaginal n = 2, total abdominal n = 9, Abdominal radical due to malignancy n= 5, Total Laparoscopic n = 4, total vaginal n = 2, abdominal radical with radiotherapy n = 1)
							Endometriosis surgery with partial cystectomy n = 2
							Vaginal reconstruction for genital malformation n = 1
							Ovarian cancer surgery with partial cystectomy n=1
Sharma et al^19^ 2016	Retrospective case series	India	Supratrigonal	Latzko repair	8	41.7 years (25-60)	Hysterectomy (vaginal n = 1, vaginal and cystocele repair n = 1, abdominal n = 6)
Kumar et al^22^ 2019	Retrospective	India	Supratrigonal	Vaginal repair	15	_	_
Lo et al^13^ 2019	Retrospective case series	Taiwan	Supratrigonal	Vaginal repair	8	50.25 years (43-65)	Abdominal hysterectomy n = 5
							Laparoscopic hysterectomy n=3
Luo and Shen^1^^4^ 2019	Cross-sectional observational analysis	China	Apical	Modified Latzko technique	108	47 years (22-77)	Hysterectomy (for a malignant condition n = 38, for a benign condition n = 64)
							Others n=6
Kizilay et al 2020^11^	Retrospective	Türkiye	Supratrigonal	Latzko repair	_		_
Panaiyadiyan 2021^6^	Cross-sectional observational analysis	India	Supratrigonal	Vaginal repair	44	37.4 (19-58)	Hysterectomy n = 34
							LSCS^*^ n = 7
							Traumatic n = 3

VVF, vesicovaginal fistulae.

*Lower segment caesarean section.

**Table 3a.** Quality Assessment of Studies

	Clearly Stated Aim	Inclusion of Consecutive Patients	Endpoints Appropriate to the Aim of Study	Follow-Up Period to the Aim of Study	Loss to Follow-Up Less Than 5%	Total Score
Ansquer et al^17^ 2006	2	0	2	2	2	8
Chigbu et al^15^ 2006	2	2	2	0	0	6
Dorairajan et al^5^ 2008	2	2	2	2	2	10
El-Lamie^18^ 2008	2	1	2	1	1	7
Lewis et al 2009	2	2	2	1	1	8
Zambon et al^16^ 2010	2	0	2	1	0	5
Rajamaheswari et al^12^ 2012	2	2	2	2	2	10
Lee et al^7^ 2014	2	2	2	2	2	10
Reisenauer^21^ 2015	2	1	2	0	0	5
Sharma et al^19^ 2016	2	0	2	2	2	8
Kumar et al^22^ 2019	2	1	2	0	0	5
Lo et al^13^ 2019	2	1	2	2	2	9
Luo and Shen^14^ 2019	2	2	2	2	2	10
Kizilay et al. 2020^11^	2	2	2	0	0	6

**Table 3b.** Quality Assessment of Studies Having Prospective Data Collection

	Clearly Stated Aim	Inclusion of Consecutive Patients	Prospective Collection of Data	Endpoints Appropriate to the Aim of Study	Unbiased Assessment of the Study Endpoint	Follow-Up Period Appropriate to the Aim of Study	Loss to Follow-Up Less Than 5%	Prospective Calculation of the Study Size	Total Score
Umoiyoho et al^20^ 2012	2	2	2	2	0	2	1	0	11
Panaiyadiyan et al^6^ 2021	2	2	2	2	0	2	2	0	12

**Table 4.** Outcomes of Studies

Study	Duration Between Fistula Onset and Repair Mean (range)	Interposition Graft	Fistula Size Mean (cm) (range)	Operative Time Mean (range)	Mean Blood Loss /Transfusion Required?	Hospital Stay Mean (range)	Postoperative Catheter Mean	Complications	Follow-Up Mean	Success Rate	Quality of Life Outcomes
Ansquer et al^17^ 2006	7.5 weeks (6-1064)	_	0.12 (0.2-4)	62 min (20-110)	_	6 days (2-12)	Foley catheter: 13 days (7-30)	UTI n = 1	19 months (1-72)	n = 11 (100%)	_
Chigbu et al^15^ 2006	Mean +/− SD 6.9 months +/- 1.3	_	2.2 (0.5-4)	_	No	16.8 days (10-25)	_	None	_	n = 21 (77.8%)	_
Dorairajan et al^5^ 2008		_	Range: 0.2 to 1	Range: 1.5-3 hours	240 mL (100-300)/ No	More than 2 weeks	Urethral catheter was removed after 2 days and suprapubic catheter was removed after 2 weeks following a voiding trial	Fever and UTI in 1 patient	Minimum follow-up of 2 years	100%	8 patients had resumed sexual activity without discomfort
El-Lamie^18^ 2008		Martius flap in cases with adjuvant radiotherapy n = 3	<3	_	_	_	_	_	_	n = 15	_
Lewis et al. 2009		_	_	_	_	_	_	_	_	73%	_
Zambon et al^16^ 2010		Yes n = 13	_	_	_	_	_	None	_	100%	_
Rajamaheswari et al^12^ 2012		Peritoneum n = 21	1.2	_	No	_	_	Bowel injury n = 1	_	n = 32 (94.1%)	No reported case of dyspareunia, increased urinary frequency, or urgency
		Omentum n = 5									
		Martius flap n = 4									
		Pre rectal fat n = 1									
Umoiyoho et al^20^ 2012		_	_	_	_	_	_	_	_	n = 28 (100%)	_
Lee et al^7^ 2014		_	_	_	_	_	_	_	Minimum 6 months	n = 32 (96.97%)	_
Reisenauer^21^ 2015		Martius flap n=12	1.27 (0.3-4.5)	_	_	_	_	_	_	100%	_
		Bioimplant interposition n = 1									
Sharma et al^19^ 2016	37.7 months (4-96)	_	0.5 (0.2-1)	60 min (45-90)	_	14 days (8-17)	Foley catheter: 14 days (14-17)	_	Range 3-8 months	100%	_
Kumar et al^22^ 2019	_	_	_	_	_	_	_	_	_	n = 10 (66.67%)	_
Lo at al^13^ 2019	124.5 days (30-210)	_	0.88 (0.5-1.5)	111.5 minutes	101.88 mL (15-300)	5.36 days (4-8)	Foley catheter: 14 days (10-21)	SUI n = 2	42.75 days (6-120)	n = 6 (75%)	UDI-6^#^ Mean (range) 9 (6-13)
								UTI n = 1			IIQ-7^$^ Mean (range) 8.75 (6-14)
Luo and Shen^14^ 2019	3.4 months (3-12)	_	< 2 n = 106 >=2 n=2	_	_	Mean +/− SD 1.2 days +/−0.3	Foley catheter removed after 4 weeks	Fever n = 6 Hematuria n = 3 Vaginal bleeding n = 3 No Clavien class 2 or greater complications	40.7 months (12-84)	92.6% n = 100 initial failure of all 8 patients was cured after a second repair with the same technique	PGI-I* much better in 46.3%, very much better in 53.7%
											Sexual discomfort in none
Kizilay et al. 2020^11^	_	_	_	_	_	_	_	None	_	n = 2 (16.67%)	_
Panaiyadiyan^6^ 2021	22.3 months (4-122)	_	1.184 (0.5-2)	101.95 minutes (70-150)	91.36 mL (40-155)	4.34 days (0-9)	Foley catheter: 22.5 days (20-26)	2 patients developed UTI and 1 had fever	27.27 months (10-65)	The study included only successful repairs	Sexual dysfunction; 6 patients had an FSFI** score ≤26.5 while 4 patients avoided intercourse (3 for fear of leak)
											4 patients had an ICIQ-SF*** score of 4, while it was 0 for the rest
											None of the patients developed SUI while 2 patients developed UUI

^#^Urogenital Distress Inventory.

^$^Incontinence Impact Questionnaire.

*Perception Global Impression of Improvement Questionnaire.

**Female Sexual Function Index.

***International Consultation of Incontinence Questionnaire–Short Form.

SUI, Stress Urinary Incontinence.

**Table 5.** Factors Prompting Vaginal Repair of Vesicovaginal Fistulae

Study	Factors That Prompted Vaginal Repair
Ansquer et al^17^ 2006	_
Chigbu et al^15^ 2006	Method of repair chosen after an examination under anesthesia
Dorairajan et al^5^ 2008	_
El-Lamie^18^ 2008	Vaginal repair preferred due to better cosmetics and lesser morbidity, blood loss, and postoperative discomfort Abdominal repair chosen when: pelvic and vaginal anatomy did not allow adequate exposure or fistula in close proximity to the ureteric orifices or insufficient hip or knee flexibility to allow for exaggerated lithotomy position or severe vaginal scarring and induration or concerns about shortening of the vagina in patients with limited capacity of the vagina which might lead to dyspareunia
Lewis et al. 2009^24^	_
Zambon et al^16^ 2010	Vaginal repair preferred in all patients except in the following: concomitant ureteral fistula which required reimplantation or bladder augmentation or history of previous radiotherapy and presence of intense vaginal stenosis
Rajamaheswari et al^12^ 2012	Vaginal repair done when fistula was accessible because of mobile vaginal wall and relaxed pelvic floor Vaginal repair not done in the following: fistula too high and could not be reached through the vagina or vaginal mobility was restricted or ureteral reimplantation was required because of the fistula overlying ureteral orifices or a comorbid pathology dictated the need for open surgery
Umoiyoho et al^20^ 2012	Simple fistulae as judged by the authors based on a self-designed scoring system were repaired vaginally. The following were excluded: complex fistulae as judged by the authors by examination under anesthesia based on the scoring system and included the following: size > 4cm, 3 previous attempts at repair, development of severe scarring, and adhesions to the pubic bone
Lee et al^7^ 2014	Choice of route of repair was at the surgeon’s discretion based on the following: location, number, ureteral involvement, and involvement of other structures, accessibility vaginal route was mostly preferred unless the fistula was not accessible vaginally or ureteral reimplantation was required
Reisenauer^21^ 2015	Vaginal route was primarily used unless the fistula was not accessible vaginally
Sharma et al^19^ 2016	_
Kumar et al^22^ 2019	_
Lo et al^13^ 2019	Vaginal route preferred unless: the presence of concomitant ureteral injury or fistula in close proximity to the ureteric orifices or complex fistula or multiple fistulae or unyielding vagina
Luo and Shen^14^ 2019	_
Kizilay et al. 2020^11^	Vaginal repair preferred. Decision was based on location, size, and surgeon’s experience. Vaginal repair preferred for fistulae < 2 cm and lying close to the bladder neck
Panaiyadiyan^6^ 2021	Route of repair largely was at the surgeon’s discretion. The following factors also contributed to the decision: location, number, size, history of prior repair, and vaginal capacity

## Data Availability

The data that support the findings of this study are available on request from the corresponding author.
